# Brain-computer interfacing using modulations of alpha activity induced by covert shifts of attention

**DOI:** 10.1186/1743-0003-8-24

**Published:** 2011-05-05

**Authors:** Matthias S Treder, Ali Bahramisharif, Nico M Schmidt, Marcel AJ van Gerven, Benjamin Blankertz

**Affiliations:** 1 Machine Learning Laboratory, Berlin Institute of Technology, Berlin Germany; 2 Radboud University Nijmegen, Institute for Computing and Information Sciences, Nijmegen, The Netherlands; 3 Radboud University Nijmegen, Donders Institute for Brain, Cognition and Behaviour, Nijmegen, The Netherlands

## Abstract

**Background:**

Visual brain-computer interfaces (BCIs) often yield high performance only when targets are fixated with the eyes. Furthermore, many paradigms use intense visual stimulation, which can be irritating especially in long BCI sessions. However, BCIs can more directly directly tap the neural processes underlying visual attention. Covert shifts of visual attention induce changes in oscillatory alpha activity in posterior cortex, even in the absence of visual stimulation. The aim was to investigate whether different pairs of directions of attention shifts can be reliably differentiated based on the electroencephalogram. To this end, healthy participants (N = 8) had to strictly fixate a central dot and covertly shift visual attention to one out of six cued directions.

**Results:**

Covert attention shifts induced a prolonged alpha synchronization over posterior electrode sites (PO and O electrodes). Spectral changes had specific topographies so that different pairs of directions could be differentiated. There was substantial variation across participants with respect to the direction pairs that could be reliably classified. Mean accuracy for the best-classifiable pair amounted to 74.6%. Furthermore, an alpha power index obtained during a relaxation measurement showed to be predictive of peak BCI performance (*r *= .66).

**Conclusions:**

Results confirm posterior alpha power modulations as a viable input modality for gaze-independent EEG-based BCIs. The pair of directions yielding optimal performance varies across participants. Consequently, participants with low control for standard directions such as left-right might resort to other pairs of directions including top and bottom. Additionally, a simple alpha index was shown to predict prospective BCI performance.

## Background

A brain-computer interface (BCI) serves to decode user intention from brain signals, enabling a direct communication between brain and computer. Since the main target group of BCIs is patients with motor impairments, it is vital that the control of a BCI does not involve motor activity. However, this is not always the case. For instance, for the widely used Matrix speller (a.k.a. P300-speller), evidence accumulates that BCI control is efficient only when the target symbol is fixated with the eyes [1-3]. Different routes have been taken to circumvent the problem of gaze dependence. For instance, one may fall back on other sensory modalities such as spatial auditory [4,5] and tactile feedback [6]. Alternatively, one may rely on other paradigms such as motor imagery [7,8]. However, motor imagery paradigms face the problem that a subset of participants does not obtain significant BCI control, a problem that is only partially solved [9-11]. Also in the visual domain, there have been promising approaches to gaze-independent BCIs. For instance, recently, three visual gaze-independent spellers have been introduced [12]. In contrast to the Matrix speller, the selection process was broken down into two successive steps, and for the best speller, mean symbol selection accuracy amounted to about 97%. Liu et al. [13] combined a similar visual design with a visual search task and reported a peak performance of 96.3%. In another study, rapid serial visual presentation of symbols was used, with a mean symbol selection accuracy of up to 90% for selecting one symbol out of thirty [14].

Note, however, that these paradigms rely on visual stimulation. In particular, they exploit the fact that the event-related potential (ERP) associated with a visual stimulus can be modulated by attention. In the present study, we take a more fundamental approach. It has been shown that covert spatial attention shifts are accompanied by power changes in the alpha band (8-12 Hz) of the electroencephalogram (EEG) at posterior electrode sites [15]. Therefore, rather than measuring the effects of attention on the neural response to visual stimulation, we directly tap the neural process underlying covert attention shifts. This approach has several advantages over conventional paradigms based on ERPs. First, continuous visual stimulation, which can be tedious and irritating especially in long BCI sessions, is superfluous. Second, for some application domains such as spatial navigation, it seems more intuitive to shift attention to the desired location rather than to perform a task such as counting the occurrences of a flashing target. Third, a BCI based on changes in oscillatory alpha activity potentially allows for asynchronous control. That is, the user initiates a covert attention shift whenever he or she wants to issue a command, whereas in an ERP paradigm, the user has to adhere to the pace and timing of the visual stimulation sequence.

**Figure 1:**
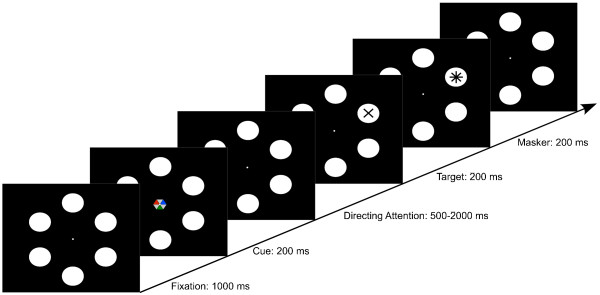
**Covert attention task**. After 1000 ms, a cue in form of a hexagon appeared. Participants had to attend to either the blue, red, or green face of the hexagon, and they had to covertly shift attention to the disc the face was pointing at. After a variable amount of time (500-2000 ms), a target ('+' or '×') appeared, followed by a masker ('*'). The participant indicated the perceived symbol by means of a button press with the right or left hand.

Kelly et al. suggested that the alpha paradigm may indeed be a feasible input modality for EEG-based BCIs [16]. Participants were instructed to deploy covert spatial attention to a target that was located either left or right of the fixation point. Offline classification showed that it is possible to discern attention shifts to either direction based on modulations of the posterior alpha rhythm. However, one of the caveats of this study was that the authors used targets flickering in different frequencies. Since the flickering might interact with the deployment of attention, it is unclear how these results transfer to a paradigm without continuous visual stimulation. Recent studies using magnetoencephalography (MEG) mapped out multiple directions of attention shifts. It was shown that shifts to multiple spatial directions, including top and bottom, yield distinctive patterns of alpha modulation [17] that can be reliably classified [18,19]. Follow-up studies investigated the role of stimulus eccentricity [20] and showed that arbitrary directions can be decoded [21]. However, it remained unclear whether the results from MEG transfer to EEG. After all, the former has a substantially higher spatial resolution which allows for a more accurate estimate of the topographical distribution of alpha power. Regarding practical application, however, an EEG-based solution is desirable due to its lower cost, portability, and the possibility to use it in a home environment. The aim of the present study was to bring together these strands of research on visual alpha based BCIs. Expanding on the work by Kelly et al. [16], we investigated whether attention shifts to directions other than left-right would also induce distinctive patterns of alpha modulation. To this end, we conducted an offline experiment wherein eight healthy participants had to shift covert spatial attention to one out of six possible target directions while strictly fixating the center of the display (see Figure 1). After a variable amount of time (500-2000 ms), a symbol (either '+' or '×') appeared on one of the six targets and participants had to indicate which one it was by pressing one of two buttons. Participants were instructed to respond as fast as possible. In 80% of the trials, the symbol appeared on the attended disc (valid condition), whereas in 20% of the trials, the symbol appeared on one of the other five discs (invalid condition). This was intended to control whether participants shifted attention to the cued location, since the reaction times should be shorter when the target appears at an attended location than when it appears at an unattended location. Refer to the methods section for more details. We will first report the behavioral results. Subsequently, we address neurophysiology and classification data. We then expand on the classification data by investigating contributions of left hemisphere versus right hemisphere electrode sites. Finally, we introduce a predictor of BCI performance based on the alpha rhythm during relaxation. Preliminary results of this study have been presented at a conference [22].

## Results and discussion

### Behavioral results

Overall response accuracy was 86.62% ± 8.46% SEM. The accuracies in the valid and invalid condition were compared using a paired-samples t-test and found to be not significantly different (*p *= .199). In contrast, the geometric means of the reaction times were significantly smaller in the valid condition than in the invalid one (*t *= 4.49, *p *< .01), indicating that the participants attended correctly the cued positions (valid: 719 ms ± 51 ms SEM; invalid: 881 ms ± 76 ms SEM).

We repeated the analysis on the subset of trials wherein the target latency was 2000 ms, since only this subset was used for neurophysiological analysis and classification (see next paragraph). For this subset, overall response accuracy amounted to 87.2% ± 8.6%. The accuracies in the valid and invalid condition were not significantly different (*p *= .233). The geometric means of the reaction times were significantly smaller in the valid condition than in the invalid one (*t *= 3.92, *p *< .01; valid: 742 ms ± 55 ms; invalid: 896 ms ± 84 ms).

### Neurophysiology

**Figure 2:**
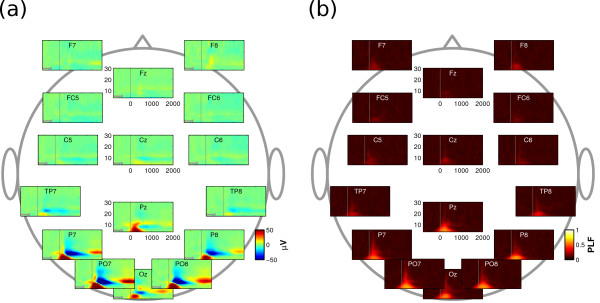
**Grand average wavelet spectra**. In each time-frequency plot, the interval of -800 to 2000 ms relative to cue onset (vertical line) is depicted on the x-axis. Morlet wavelet center frequencies, ranging from 4 to 30 Hz, are depicted on the y-axis. Color signifies wavelet amplitude in (a) and the phase-locking factor in (b). (a) At posterior electrode sites, three neurophysiological events can be observed, namely an early synchronization in the delta and theta bands, followed by a desynchronization and subsequent synchronization in the alpha band. (b) Phase-locking factor (PLF), specifying the amount of phase-locking to stimulus onset. Only the early synchronization in the delta and theta bands is phase-locked to stimulus onset. This supports the idea that the early component reflects the processing of the visual cue, while the alpha (de)synchronization is associated with the deployment of covert visual attention.

For neurophysiological analysis and classification, we used the subset of trials with a 2000 ms target latency. Trials with shorter target latencies were not considered since they were only intended to stimulate participants to shift their attention immediately after cue onset. In the former trials the whole 2000 ms contain the shift and maintenance of attention to the target without any external stimulus. The spatial resolution of the EEG data was enhanced using a current source density estimate [23]. Figure 2 depicts grand-average wavelet spectra for a subset of scalp channels, averaged over all six directions and all participants. In Figure 2a, wavelet coefficients were determined for single trials and then averaged over all trials and participants. Note that wavelets are acausal filters, that is, post-stimulus activity can leak into the pre-stimulus baseline. Therefore, baseline-correction was performed on the -800 to -419 ms interval, as indicated by the grey bar in each subplot. Choosing -419 as upper bound prevented post-cue activity from leaking into the baseline because it corresponds to half the width of the widest wavelet. The spectra show three distinct neurophysiological events preponderating at posterior electrode sites, with little event-related activity at other electrode sites. First, a synchronization in the delta and theta bands peaking at 200-300 ms. Second, a desynchronization in the alpha band peaking roughly at 500 ms. Third, a subsequent late synchronization alpha band evident from about 1500 ms. In Figure 2b, the phase-locking factor (PLF) was calculated by first normalizing wavelet coefficients to unit magnitude, averaging over epochs and then determining the magnitude of the result [24]. Only the first of the events depicted in Figure 2a displays phase-locking with stimulus onset, suggesting that the early delta and theta activity is caused by ERPs that reflect the visual processing of the cue. In line with the literature (e.g., [15,18,25]), we found that an alpha desynchronization and a subsequent synchronization indexes shifts of covert visual attention.

For each participant, and for each of the fifteen possible pairs of directions, we performed binary classification using logistic regression and computed classification accuracy under a ten-fold cross-validation scheme [26,27]. In order to reduce sensitivity to overfitting, an L2 regularizer was added to the classifier's objective function [28]. This regularizer is controlled by a regularization parameter that effectively shrinks the estimated regression coefficients towards zero. In order to determine the optimal regularization parameter a grid search was performed and the smallest parameter value was chosen that gave highest accuracy as computed with five-fold cross-validation using just the training data of the outer ten-fold cross-validation. Subsequently, the classifier was retrained using all training data in order to test the classifier on the test data. Significance levels were calculated by comparing classification outcomes with an assignment of all outcomes to the majority class using a McNemar test [29]. For comparative purposes, classification was repeated using L1 regularization, but it was found to yield lower classification accuracy than L2 regularization.

Since alpha power peaks over occipital electrodes sites, the subset of electrodes comprising PO3,4,7-10, and Oz,1,2, was selected as input to the classifier. We focused only on alpha synchronization, because the preceding alpha desynchronization did not show distinctive patterns for the different directions. For each electrode, a single spectral feature was extracted by estimating bandpower in the alpha range (8-12 Hz) for the 500-2000 ms interval using the Welch method. In other words, the interval was split into 8 segments with 50% overlap between segments. Each segment was windowed using a Hamming window. Spectral power was estimated in each segment and then averaged across segments. During cross validation, for each participant, data was normalized to have zero mean and a standard deviation of one in the training set of the outer fold.

**Figure 3:**
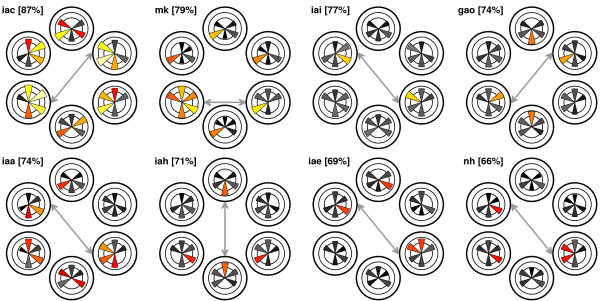
**Binary classification results for each of the eight participants and for each pair of directions**. Peak accuracy for the best-classifiable pair of directions is given in brackets after the participant code; this pair is also indicated by a double arrow. Classification scores are depicted for all binary pairings of directions. For each participant, the data consists of six polar plots placed at spatial locations analogous to the locations used in the experiment. Each polar plot contains five pie slices depicting classification accuracies between the location of the plot and each of the five other possible directions. Classification accuracies that are significantly different (*p *< .05) from chance level (50%) are given as yellow-red pie slices, non-significant accuracies are shaded grey. Both the length of a pie piece and its color indicate classification accuracy (lighter color for higher accuracy, darker color for lower accuracy). For instance, for participant iai, only the top-left and the bottom-right directions could be differentiated from each other significantly.

Mean accuracy for the best pair of directions was 74.6% ± 2.3%. Figure 3 depicts the classification accuracy for each participant and for each pair of directions. Colored pie pieces represent directions that were significant under a significance threshold of 0.05. Moreover, for three participants (iac, mk, and iaa) results were highly significant (*p *< .001). The figure reveals large individual differences. In particular, the pair of directions yielding the best classification performance varied substantially across participants. In most cases, some combination of left and right directions yielded the best classification performance.

**Figure 4:**
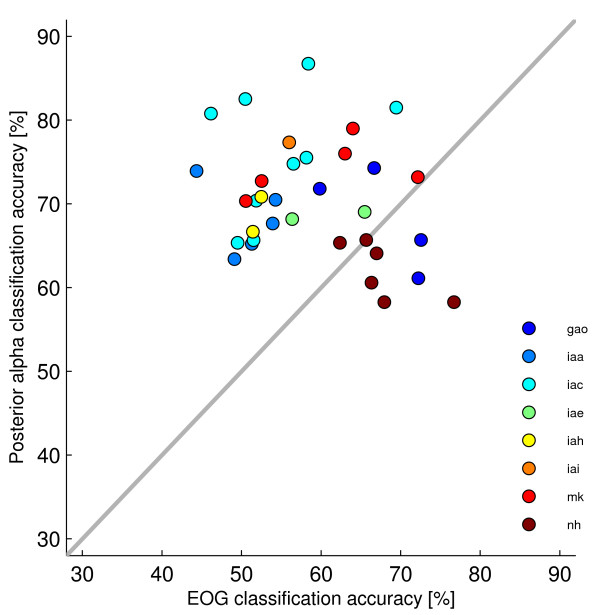
**Classification accuracies using EOG versus EEG**. For each participant, only those direction pairs are depicted which yielded significant classification results based on EEG and/or EOG. Notably, high accuracy for EEG-based classification usually comes with low accuracy for EOG-based classification, and vice versa. This suggests a dissociation between EEG- and EOG-based classification.

To check for confounds, we applied a logistic regression classifier on the time series obtained with two bipolar EOG channels. Highly significant classification performance (*p *< .001) was obtained for only one direction in one participant. Under a significance threshold of 0.05, EOG data alone was not sufficient to obtain significant classification outcomes for three participants. For participants mk, iae, and iac, only one pair of directions was classifiable. For participant gao, this was the case for two pairs of directions (top-right versus top-left and bottom-right versus bottom-left). For participant nh, five pairs of directions could be classified. Note that the latter participant yielded the worst classification results on the EEG data (see Figure 3), which suggests a dissociation of the processes underlying EOG activity and posterior alpha activity. In line with this, the scatter plot shown in Figure 4 makes clear that there is no significant correlation (*r *= .029, *p *= .75) between the classification outcomes obtained using either EEG or EOG measurements.

### Left hemisphere versus right hemisphere contribution

**Figure 5:**
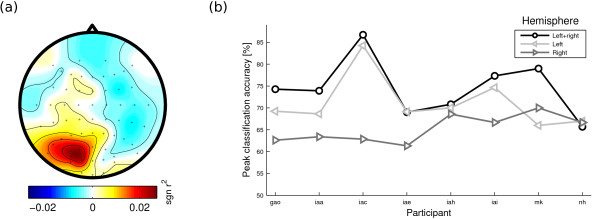
**Contribution of left and right hemispheres to classification success**. (a) Point-biserial correlation coefficient contrasting spectral power for shifts to right versus left directions. The *sgn r*^2 ^is peaking over the left hemisphere only. No differential effect is observed over the right hemisphere. (b) Peak classification accuracy when only left hemisphere electrodes, only right hemisphere electrodes, or both sets are used for classification. For illustrative purposes, data points belonging to the same electrode montage have been connected by lines. The graph suggests that left hemisphere electrodes yield a higher performance than right hemisphere electrodes.

There is evidence that the left and the right hemisphere do not contribute equally to shifts of visual attention [30]. In particular, the left hemisphere mainly supports shifts of attention in the contralateral (right) hemifield, while the right hemisphere is involved in attention shifts in both hemifields. To investigate whether this asymmetry applies to the present data as well, we pooled over both left and both right directions and estimated alpha power in the classification interval (500-2000 ms) for both directions. Subsequently, we calculated the signed square of the point-biserial correlation coefficient *sgn r*^2 ^(see, e.g., [31]), contrasting shifts to right directions with shifts to left directions. The results are depicted in Figure 5a. In line with the literature, alpha power is higher at left hemisphere electrode sites when attention is directed to the right than when attention is directed to the left. For right hemisphere electrode sites, alpha power does not differ significantly for shifts to right and shifts to left directions.

As a consequence, one would expect an asymmetric impact of electrode position on BCI performance, with left hemisphere electrodes contributing more to classification success than right hemisphere electrodes. As Figure 5b suggests, this is indeed the case. For most participants, classification on left hemisphere electrodes yields better scores than classification on right hemisphere electrodes. Nevertheless, taking into account both hemispheres usually improves performance, suggesting that right hemisphere electrodes add independent information. To compare these three conditions quantitatively, we performed a 1-way analysis of variance (ANOVA) on the peak performances in the three conditions. We found a significant effect of the electrode subset (left, right, or both hemispheres) on BCI performance (*F *= 6.11, *p *< .01).

Tukey-Kramer post-hoc tests revealed that classification using both hemispheres gives better accuracy than classification using left hemisphere only. The other contrasts were not significant.

### Alpha rhythm based predictor of BCI performance

In light of the availability of numerous BCI systems and the fact some users do not obtain significant BCI control, prediction of BCI performance using simple neurophysiological indices is a topic that is gaining increasing attention [9]. Our aim was to use posterior alpha power from the resting EEG as a predictor of BCI performance. To this end, we investigated the relaxation data recorded prior to each experiment. We considered the epochs wherein participants relaxed with eyes closed. After current source density filtering [23], the spectral peak in the 8-12 Hz alpha range was extracted for each electrode.

**Figure 6:**
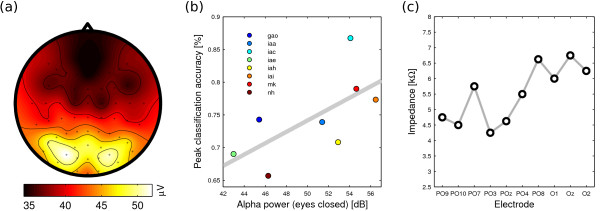
**Prediction of BCI performance based on the alpha rhythm**. (a) Spatial distribution of alpha during relaxation with eyes closed. Alpha amplitude is highest over the electrode subset that was used for classification (i.e., PO3,4,7-10, and Oz,1,2), with absolute peaks at electrodes PO7 and PO8. (b) Correlation between alpha power at electrode pair PO3-PO4 and peak classification accuracy (*r *= .66). The grey line gives a linear fit. (c) Mean impedances across participants show lower impedance for PO3-PO4 than for PO7-PO8. This possibly explains why the former pair is more predictive of BCI performance than the latter.

Figure 6a shows that alpha energy dominates at parieto-occipital electrode sites. Consequently, we considered pooled alpha power of symmetric electrode pairs at parieto-occipital sites as a predictor. For electrode pair PO3-PO4, a correlation of *r *= .66 (*p *= .07) was found, see Figure 6b. For electrode pair PO7-PO8, correlation drops (*r *= .54; *p *= .17), despite the higher absolute power. We suppose that this might stem from the fact that mean impedance was lower for PO3-PO4 than for PO7-PO8, yielding a cleaner EEG signal (Figure 6c).

## Discussion

Shifts of covert visual attention induce changes in alpha power over posterior electrode sites. Initial analyses revealed that an early desynchronization was of little discriminative value regarding the direction of attention shifts. We believe that this early desynchronization may be related to the preparation of covert attention shifts. A subsequent synchronization, however, yielded distinctive topographic patterns for the different directions and served as a basis for classification.

Using regularized logistic regression, significant binary classification performance was obtained for each participant, with a mean accuracy of 73.65% for the best pair of directions. A classification accuracy of 70% was proposed as performance threshold above which BCI performance can be considered as robust [32,33]. In the present study, six participants had a peak performance above 70%, and two participants had a performance that was slightly lower (66% and 69%). Interestingly, this figure is close to the accuracy obtained in earlier MEG studies, in spite of the significantly higher spatial resolution of MEG as compared to EEG [18,21]. This suggests that changes in alpha power following covert attention shifts are rather broadly distributed in visual cortex and, hence, can be mapped with sufficient precision using EEG.

Mean classification accuracy obtained in the present study is similar to the accuracy obtained by Kelly et al. [16]. However, there are significant methodological differences. First, Kelly et al. used visual stimulation in form of two flickering stimuli. It is unclear how the flickering affects the ease of deploying attention to the visual periphery. Second, just as we did, Kelly et al. used cross-validation to estimate classification performance. However, epochs were partly overlapping. In other words, the training set (used to train the classifier) partly contained information about the test set (used to verify the classifier), which might have led to an overestimation of classification accuracy.

The pair of directions yielding the highest classification performance varies considerably across participants (see grey double arrows in Figure 3). For all but one participant, locations at opposite sides of the fixation point yield optimal performance. Furthermore, for seven participants, highest performance is achieved with a combination of left and right directions. Mostly, this combination also has a vertical offset (i.e., top-left combined with bottom-right, or bottom-left with top-right). For the other participant (iah), peak performance is achieved when attention is shifted in the vertical direction. This indicates that left versus right is not necessarily the optimal pair of directions. Therefore, participants with low control for these directions may resort to other pairs of directions including top and bottom.

Furthermore, we found an asymmetry regarding the contribution of electrode sites to classification success. In particular, left hemisphere electrodes contributed more to classification success than right hemisphere electrodes. This is in line with evidence that the left hemisphere supports mainly attention shifts to the right hemifield, while the right hemisphere is involved in attention shifts to both the right and the left hemifields [30].

### Prediction of BCI performance

Due to the proliferation of BCI research in the last decade, there exists now a wide palette of BCI systems. However, there is no *a priori *criterion for assigning a particular BCI system or a particular input modality (such as event-related potentials or sensorimotor rhythm) to a new BCI user, despite the fact that there is high variability across users regarding the efficiency of particular BCI paradigms. As a result, BCI users might use a system that does not yield optimal performance. This problem is aggravated by the fact that a non-negligible proportion of participants fails to exhibit significant BCI control. For paradigms based on the modulation of the sensorimotor rhythm (SMR), this proportion amounts to 15-30% of the participant population [9].

Consequently, there is growing need for efficient screening procedures that allow for the estimation of prospective BCI performance. To be useful, screenings should be obtained within few minutes using a simple paradigm, in order to prevent a tedious and, upon failure, frustrating calibration procedure. For instance, Blankertz et al. showed that the *mu *rhythm generated in motor cortex is predictive of BCI performance in a motor imagery paradigm [9]. The predictor was obtained from a 2 minutes measurement during which participants were instructed to relax with eyes open. It showed a correlation of *r *= .53 with BCI performance.

In a similar fashion, we developed a predictor of BCI performance based on a 3 minutes relaxation measurement with eyes closed. For each participant, the invidiual alpha peak was extracted and power was combined for electrodes PO3 and PO4. A correlation of *r *= .66 was found between the alpha index and peak BCI performance, suggesting that BCI performance can be predicted from a simple resting EEG measurement.

## Conclusions

The present study suggests that modulations of alpha power associated with covert attention shifts form a viable input modality for EEG-based BCIs. Furthermore, an alpha index obtained during a short relaxation measurement can predict prospective BCI performance. Analogous to the motor imagery paradigm, where different types of imagery (e.g., movement of left hand, right hand, and foot) are tested preliminary and the best pair is chosen, eligible participants might then be screened for different directions of covert attention shifts. In order to maximize performance, the BCI would be tuned to the pair of directions that provides the best classification accuracy.

## Methods

### Participants

Eight healthy volunteers (seven male, one female), aged 18-27 years, participated in this study. One of the participants was a co-author (NS), all others were naïve with respect to BCIs. All had normal or corrected-to-normal vision. All participants gave written consent and the study was performed in accordance with the Declaration of Helsinki.

### Task and Stimuli

The main experiment was preceded by a six minutes relaxation measurement. It comprised two alternating phases, namely an eyes closed phase, wherein participants simply relaxed and closed their eyes, and an eyes open phase, wherein they observed a small polygon on the computer screen changing shape and color. The duration of each phase was 15 s with 2 s breaks in between, and the total measurement lasted for about 6 minutes.

In the main experiment, participants performed a cued visual attention task. The course of a trial is depicted in Figure 1. First, a white central fixation dot surrounded by six white target discs was presented. The discs had a size of 3.27° of visual angle and they were presented at an eccentricity of 9° from the fixation dot. A cue appearing for 200 ms in the center of the screen indicated the target location. Participants had to shift attention to the cued disc while strictly fixating the central dot. Instead of arrows, we used an omnidirectional cue to reduce the danger of evoking event-related potentials specific to the direction of the cue. The cue was a hexagon with each of the six faces pointing to one of the target discs. Three of the faces were grey and the other three were colored blue, red, and green, respectively. One of these colors was used as target indicator, that is, the participant had to covertly direct and maintain attention to the disc to which this color was pointing. The use of one of the three colors as target color was counterbalanced across participants. After a variable duration (500-2000 ms) the target appeared for 200 ms in the disc as either a '+' or a '×'. Participants indicated which symbol they had perceived by pressing with their thumb on one of two buttons lying in the palm of the right and left hands. Two different targets had been chosen to reduce readiness potentials for pressing a button, as suggested by [17]. After 200 ms, a star-shaped masker (' *') was presented at the target location for 200 ms in order to prevent an afterimage of the target and thereby increase task difficulty.

Each participant completed 600 trials in six blocks of 100 trials with two-minute breaks between blocks. Cues were valid in 80% of the trials. In the other 20% of the cases, the target appeared at a different random location. The target symbol was randomly chosen, with equal chances for '+' and '×'. Target latency (i.e., the time between cue onset and target onset) was 2000 ms in 50% of the trials. To ensure that the participants shift their attention immediately after the appearance of the cue, 30% of the trials featured a short target latency of 500 ms. In the remaining trials, the target latency was randomized between 500 ms and 2000 ms in order to ensure that attention is sustained continuously until target appearance.

### Apparatus

EEG was recorded from a Brain Products (Munich, Germany) 64 channel actiCAP, digitized at a sample rate of 1000 Hz, with impedances kept below 20 kΩ. We used electrodes Fp2, AF3,4, Fz, F1-10, FCz, FC1-6, T7,8, Cz, C1-6, TP7,8, CPz, CP1-6, Pz, P1-10, POz, PO3,4,7-10, Oz,1,2 and Iz,1,2, placed according to the international 10-10 system and referenced against a nose reference. Additionally, an EOG electrode labelled EOGvu was placed below the right eye. Vertical and horizontal bipolar EOG channels were created by referencing Fp2 against EOGvu, and F10 against F9, respectively. Stimuli were presented on a 24" TFT screen with a refresh rate of 60 Hz and a resolution of 1920 × 1200 px^2^. The experiment was implemented in Python using the open-source BCI framework Pyff [34] with Pygame http://pygame.org. Data analysis and classification were performed with MATLAB (The MathWorks, Natick, MA, USA) using custom functions and the Fieldtrip toolbox for EEG/MEG-analysis (Donders Institute for Brain, Cognition and Behaviour, Radboud University Nijmegen, the Netherlands. See http://www.ru.nl/neuroimaging/fieldtrip).

## Authors' contributions

MT and BB conceptualized the study. NS, MT, and BB implemented the software and ran the measurements. MT prepared a first draft of the manuscript. AB, MG, and MT performed the classification and contributed the respective section in the manuscript. All authors read, revised, and approved the manuscript.
